# Characterization of the melanoma brain metastatic niche in mice and humans

**DOI:** 10.1002/cam4.45

**Published:** 2013-03-11

**Authors:** Moran Amit, Leonor Laider-Trejo, Vardit Shalom, Ayelet Shabtay-Orbach, Yakov Krelin, Ziv Gil

**Affiliations:** 1 The Laboratory for Applied Cancer Research, Tel Aviv Sourasky Medical Center, Sackler Faculty of Medicine, Tel Aviv UniversityTel Aviv, Israel; 2 The Pathology Institute, Tel Aviv Sourasky Medical Center, Sackler Faculty of Medicine, Tel Aviv UniversityTel Aviv, Israel; 3 Department of Otolaryngology Head and Neck Surgery, Rambam Medical Center, Rappaport School of Medicine, the Technion Israel Institute of TechnologyHaifa, Israel

**Keywords:** Cancer biology, clinical cancer research

## Abstract

Brain metastases occur in 15% of patients with melanoma and are associated with a dismal prognosis. Here, we investigate the architectural phenotype and stromal reaction of melanoma brain metastasis in mice and humans. A syngeneic, green fluorescence protein (GFP)-expressing murine B16-F1 melanoma clone was introduced via intracardiac injection, and was examined in vivo in comparison with human specimens. Immunofluorescence analyses of the brain metastases revealed that F4/80^+^ macrophages/microglia were most abundant at the tumor front, but rare in its core, where they were found only around blood vessels (*P* = 0.01). Similar pattern of infiltration was found in CD3^+^ T cells (*P* < 0.01). Infiltrating T cells were prominently CD4^+^ compared with CD8^+^ T cells (*P* < 0.001). Blood vessels (CD31^+^) were less abundant at the tumor front than in its center (12 ± 1 vs. 4 ± 0.6 vessels per high-power field [HPF], *P* < 0.001). In contrast, there were few vessels at the tumor front, but their diameter was significantly larger at the front (8236 μm^2^ vs. 4617 μm^2^ average cross-sectional area, *P* < 0.005). This is the first comparative analysis of melanoma brain metastases showing similar stromal reaction in murine models and human specimens. Our results validate the utility of this murine model of melanoma brain metastases for investigating the mechanism of the human disease.

## Introduction

Central nervous system (CNS) metastasis occurs in 15% of the patients with melanoma and is associated with a dismal prognosis 1. Affected patients are likely to suffer from severe neurological deficits, general deterioration, and eventually succumb to cancer within 12 months of diagnosis 2. The formation and anatomical location of metastases is determined by both tumor cell properties and host factors. Specific genes are known to be involved in tumor development, but the basis for brain metastases is largely undetermined 3. The brain may provide a unique microenvironment that is very different from that of any other part of the body. The blood–brain barrier creates a privileged immune environment, which is comprised of a range of unique cell types, including infiltrating monocyte-derived brain macrophages and microglia 4. In response to brain injury, these cells accumulate and are activated to promote neuron survival, a process resulting in the formation of gliosis 5. In addition, blood–brain barrier disruption leads to the crossing of macrophages and lymphocytes from the blood into the brain. Analysis of pathological specimens of brain metastases excised from cancer patients revealed a significant increase in the number of immunocytes in the tumor microenvironment 6. It was suggested that the brain-specific tumor microenvironment can promote metastatic growth 7. Immune cell composition within the tumor niche may also have different effects on the development and progression of metastases 8.

Systemic therapies often fail to improve a patient's survival and usually have a low response rate 9. A major impediment in drug development against melanoma metastases is the lack of reliable animal models that can predict tumor response to therapy. Relevant animal models that recapitulate the specific traits of the metastatic niche in an immunocompetent animal are needed in order to enable these lines of therapeutic research. Here, we investigated the architectural phenotype and stromal reaction of melanoma brain metastasis in mice and humans. This article describes our efforts to validate the utility of a model of murine melanoma brain metastases for investigating the mechanism of the human form of the disease. We reason that better understanding of basic immunocyte infiltration and cytotoxic potential within brain metastases will lead to improved treatment protocols in the prevention and control of melanoma brain metastases.

## Materials and Methods

### Reagents used for immunohistochemistry

The following antibodies were used: rat anti-mouse/human F4/80 (1:250; AbDSerotec, Kidlington, U.K.), rat anti-mouse/human CD3e (1:50; AbDSerotec, Kidlington, U.K.), mouse monoclonal anti-CD4 antibody (1:250; clone ab51312 Abcam Cambridge, U.K.), rabbit polyclonal anti-CD8 antibody (1:200; clone ab4055 Abcam Cambridge, U.K.), and rat anti-mouse CD31 antibody (1:100; Dianova, Hamburg, Germany). For immunofluorescent labeling, the DyLight 594-conjugated AffiniPure donkey anti-rat IgG and DyLight 488-conjugated AffiniPure donkey anti-rabbit IgG antibodies (1:200; Jackson ImmunoResearch Laboratories, PA) were used as secondary antibodies. For hydrogen peroxide labeling, an horseradish peroxidase (HRP)-conjugated polyclonal goat to mouse immunoglobulin G (IgG) (1:500; clone ab6789 Abcam Cambridge, U.K.) and a biotinylated goat anti-rabbit antibody (1:1000; clone ab6721 Abcam Cambridge, U.K.) were used as secondary antibodies. Immunofluorescent slides were covered with Dapi-Fluoromount-G (SouthernBiotech, AL). Hydrogen peroxide-labeled tissues were visualized by using DAB as a substrate (clone ab64238, Abcam Cambridge, U.K.).

### Melanoma cell line

The B16-a-GFP melanoma cell line was a kind gift from the laboratory of Guy Shakhar (Weizmann Institute, Rehovot, Israel). This murine melanoma line expresses green fluorescent protein in vivo 10, allowing the detection of graft-derived cells in the recipient mice using fluorescent histology, flow cytometry, and intravital 2-photon microscopy. Cells were grown in Dulbecco's modified Eagle's medium containing 10% fetal calf serum and 1% penicillin–streptomycin (Invitrogen, CA), and incubated in a 5% CO_2_-humidified incubator at 37°C.

### Mice and the in vivo CNS metastasis model

Wild-type C57BL/6 female (4–8-week-old) mice were obtained from Harlan Laboratories (Jerusalem, Israel). They were kept at the Animal Facilities of the Tel Aviv Sourasky Medical Center (TASMC, Tel Aviv, Israel) under the strict guidelines of the Institutional Animal Care and Use Committee-approved protocols. Animal studies were performed in compliance with all applicable policies, procedures, and regulatory requirements of the Institutional Animal Care and Use Committee, the Research Animal Resource Center of Tel Aviv University and the National Institutes of Health “Guide for the Care and Use of Laboratory Animals.” All the procedures were performed under inhalational anesthesia using 2% isoflurane, and the animals were sacrificed by CO_2_ inhalation after the procedures were completed.

A brain melanoma metastasis model was established as described previously by Fidler et al. 11. Briefly, forty 4- to 8-week-old mice (Harlan Laboratories, Jerusalem, Israel) were anesthetized with inhalational isoflurane for all procedures. After restraining the animals on a pad, their chests were shaved and sterilized with alcohol. Under the guidance of an ultrasound Doppler (Vevo 2100, VisualSonics, SonoSite, Toronto, Ontario, Canada), the left cardiac ventricles were slowly injected with 100 μL of cell suspension at a concentration of 10^5^ or 2 × 10^4^ cells/100 μL over 1 min. The mice were then awakened and monitored for established signs of distress and discomfort, including development of cachexia (as defined by a loss of 20% of original body weight), limb paresis, or paralysis and the inability to move and reach for the food source. After 8 weeks, all the surviving animals were sacrificed and systematically examined for the presence of metastases. Tumor volume (V) was calculated as V = ∏xab^2^/6, where *a* is the longer and *b* the shorter of two perpendicular tumor diameters, as measured with calipers.

### Characterization of human and mice CNS metastasis microenvironment

For immunofluorescence staining and imaging of brain metastases, samples from the mice brain were obtained, fixed in 4% paraformaldehyde, dehydrated in alcohol, cleared in xylene, and embedded in paraffin. Four-micron sections were stained with hematoxylin and eosin using established protocols. The human specimens were randomly selected CNS (i.e., brain parenchyma) melanoma metastases from patients who had been identified through a search of the TASMC pathology department's archive after approval by the Institutional Review Board. Archival samples of CNS melanoma metastases from tumors that had been surgically resected between 1 January 2009 and 31 December 2009 at TASMC were selected for analysis. Only samples from previously untreated patients with available paraffin-embedded primary tumor tissue were obtained.

For immunohistochemistry, the tissue sections were deparaffinized in xylene and rehydrated with decreasing concentrations of alcohol. Endogenous peroxide was blocked with hydrogen peroxide and antigens were retrieved by using 0.01 mol/L sodium citrate (pH 6.0) for 1 min in a pressure cooker. After blocking the preparations in the appropriate normal serum, they were incubated overnight with primary antibodies at 4°C. The slides were then washed twice with TBS–TritonX 0.025% for 5 min and incubated with secondary antibodies at room temperature for 1 h at 1:200 dilution. Immunofluorescent slides were then covered with Dapi-Fluoromount-G (SouthernBiotech, AL) and examined by fluorescence microscopy (cell^R – Imaging Station; Olympus, Hamburg, Germany). Hydrogen peroxide-labeled tissues were visualized by using DAB as a substrate (clone ab64238, Abcam Cambridge, U.K.).

### Image analysis

Identical exposure times and image settings were used for each experiment. Images were analyzed using ImageJ software (http://rsb.info.nih.gov/ij). For each specimen, two images per region of interest were captured at 20× magnification for quantification. The regions of interest were defined based on Dapi-Fluoromount-G fluorescence and were outlined by “freehand.” Two independent approaches were used to quantify the observed immunohistochemical signal. First, we manually counted cells per high-power field (HPF, 20× objective; each field measuring 433.3 × 350 μm) using a cell counter plug-in of ImageJ software. Then, green, red, and blue channels were isolated using ImageJ software, and a threshold was determined for quantification of signal intensity. Each pixel in the identified regions was assigned a fluorescence intensity value (based on a scale from 0 to 255). The average pixel density (mean fluorescence intensity, MFI) within the selected area was calculated. The average pixel density of the two images was calculated.

For angiogenesis analysis, three random 0.151 mm^2^ fields at 200× magnification within tumor core and tumor front were captured using a fluorescence microscopy charge-coupled device color camera (cell^R – Imaging Station; Olympus, Hamburg, Germany). The vessels were quantified using ImageJ software. A structure was classified as a vessel when single endothelial cells or clusters of endothelial cells were positive for CD31 12. The presence of blood cells or fibrin without any detectable endothelial cells was not sufficient to define a microvessel. Vessels with muscular walls were not counted. Two independent observers (M. A. and Z. G.) counted the number of microvessels in the histological field using the ImageJ program. Microvessel density (MVD) was then assessed according to Weidner et al. 13. The MVD of the specimen was estimated as a mean of MVD in the three histological fields.

For calculation of the vessel's cross-sectional area, CD31-positive vessels with lumens were selected for each field. Each vessel was manually outlined, and the cross-sectional area was measured within the same fields as before, using a 200× magnification. The mean cross-sectional area values were calculated. To ensure the reliability of angiogenesis determination, inter- and intraobserver error was checked by the Spearman rank correlation coefficient. The tissue specimens were analyzed independently by two investigators who were unaware of the specimen's data. The vessels were counted twice within 1 month to detect any intraobserver error: the slides were reassessed by both investigators if an error had occurred.

### Statistical analysis

Unless otherwise indicated, the data are presented as mean ± SEM. All other group differences were evaluated by two-tailed unpaired Student's *t*-test (JMP, SAS, NC). Survival data were analyzed by Kaplan–Meier log rank tests (OriginPro, OriginLab, MA). Fisher's exact test was used to compare proportions of mice with CNS metastasis (JMP, SAS, NC). *P* values less than 0.05 were considered significant.

## Results

### Establishing brain metastases of a green fluorescence protein-expressing melanoma model

**Figure 1:**
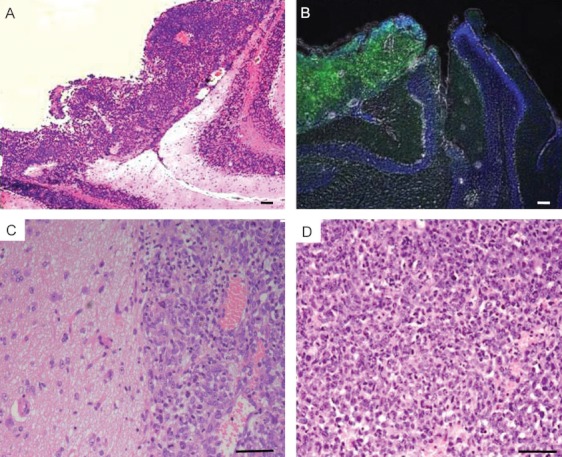
Histological characterization of brain metastatic melanoma. (A) Meningeal metastasis of mouse *B16-GFP* melanoma (hematoxylin and eosin 4×). (B) Immunofluorescent staining with anti-GFP Ab (green) and Dapi-Fluoromount-G (blue). (C) High-power field (20×) of tumor front. The tumor front was defined morphologically as the intersection of normal brain parenchyma with the tumor. (D) High-power field (20×) of the tumor core. Cores are considered as being the center of tumor where no normal brain tissue is visible within a single high-power field. All scale bars are 100 μm.

We first sought to evaluate the metastatic potential of the mouse cell line using intracardiac injections. To establish a brain metastasis, we injected 2 × 10^4^ or 10^5^ freshly dissociated B16-a-GFP melanoma cells under ultrasound guidance into the left ventricle of C57BL/6 mice. These mice were followed daily for 8 weeks, and sacrificed within 8 weeks when their performance status was poor. The median survival time after the intracardiac injection of 2 × 10^4^ and 10^5^ cells was 26 and 29 days, respectively (*P* = 0.20). To assess the location of distant metastases, we used fluorescent imaging of e**-**green fluorescence protein (GFP)-expressing melanoma cells (Fig. 1). Eight animals had intracranial metastases (Fig. S1). There was equal distribution of brain parenchyma metastases and leptomeningeal metastases (four each), but the tumor volume in the meninges was significantly smaller than within brain parenchyma (21 ± 4 and 7 ± 3 mm^3^, respectively; *P* = 0.03). In addition to brain metastases, tumors also metastasized to the lung (*n* = 3), to the liver (*n* = 6), and to bone (*n* = 2).

### Immune host response to melanoma brain metastases in mice

**Figure 2:**
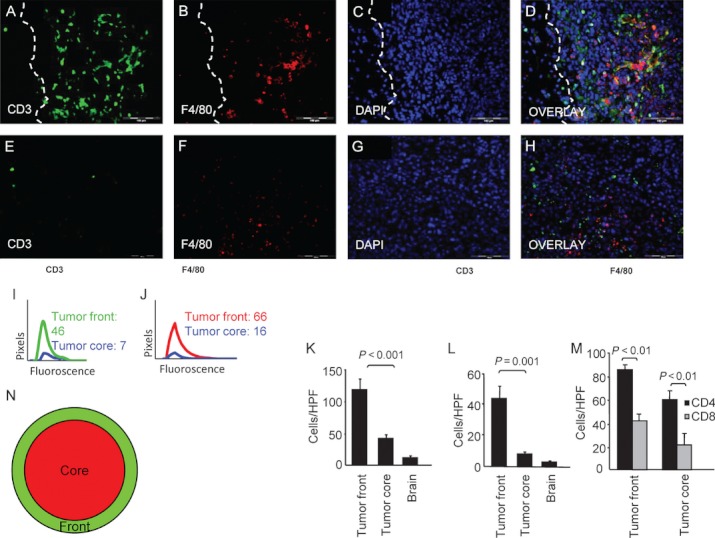
Immune infiltration of brain melanoma metastases in mice. (A–D) Immunofluorescence staining with anti-CD3 (green) and anti-F4/80 (red) Abs. Nuclei were stained with Dapi-Fluoromount-G (blue). Fluorescence analysis showed the same pattern of infiltration of CD3^+^ and F4/80^+^ predominantly at the tumor front (indicated by the dashed line). (E–H) Similar immunostaining in the tumor core, showing reduced immunocytes infiltration. (I) Curves representing quantitative image analysis of the immunofluorescence intensity of CD3^+^ and (J) F4/80^+^ cells. The numbers denote mean fluorescence intensity (MFI). Data are representative of three independent experiments (*P* < 0.05). (K) Histogram showing quantification of CD3^+^ cells (mean ± SEM; *n* = 8). (L) Histogram showing quantification of F4/80^+^ cells. (M) Histogram showing quantification of CD4^+^ and CD8^+^ cells within tumor front and tumor core (mean ± SEM; *n* = 6). (N) An illustrative depiction of the tumor regions. Red circle indicates the tumor core, and green circle the tumor front. All scale bars are 100 μm.

A principal feature of the metastatic niche is the immune host response to tumor development. Therefore, we aimed to characterize the immunocyte composition of the metastatic niche in the murine model specimens. Immunohistochemical fluorescence analyses by anti-F4/80 Ab (*n* = 8) was used to quantify the macrophages/microglia infiltrates. The macrophages/microglia were most abundant at the tumor front compared with the center of tumor (*P* = 0.001, Fig. 2). Fluorescent intensity analysis revealed significantly higher staining at the tumor front (66 ± 16) relative to its core (16 ± 5, *P* = 0.04, Fig. 2J). Similar to microglia/macrophages, CD3-positive T-cell infiltration was more prominent at the tumor front compared with its core (*P* < 0.001, Fig. 2K). Image analysis for the fluorescence intensity of CD3-positive staining showed a significantly higher signal at the tumor front than its core (46 ± 7 and 7 ± 3, respectively; *P* = 0.02, Fig. 2I). Further subtype analysis of the CD3-positive T cells revealed that these were mainly CD3^+^CD4^+^-infiltrating cells (*P* = 0.002, Fig. 2M). A higher infiltration of CD3^+^CD4^+^ cells relative to CD3^+^CD8^+^ cells was found both at the tumor front and in its core. For comparison, we evaluated the adjacent normal brain parenchyma using similar methods. Immunohistochemical analysis revealed only sparse CD3-positive and F4/80-positive cells in normal brain tissue, significantly fewer than those found in the metastatic niche (*P* < 0.001, Fig. 2).

### Immune host response to specimens of brain metastases from melanoma patients

**Figure 3:**
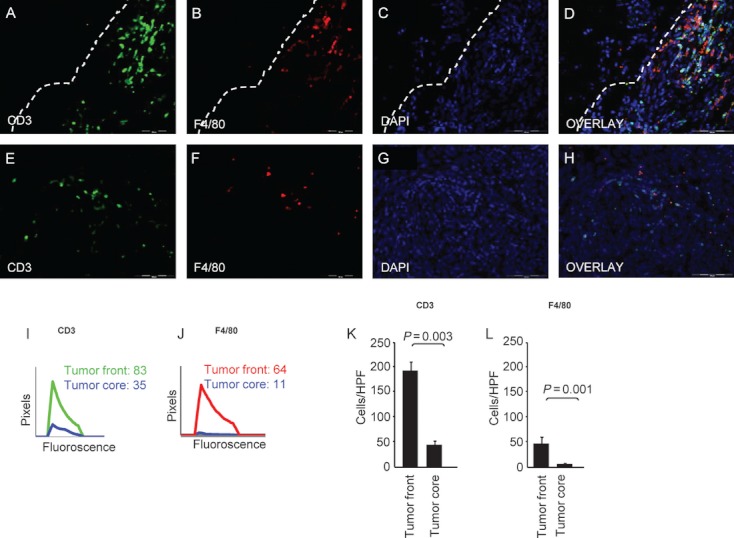
Immune trafficking of CNS melanoma metastasis microenvironment in surgically excised human specimens. (A–D) Immunofluorescence staining with anti-CD3 (green) and anti-F4/80 (red) Abs. Nuclei were stained with Dapi-Fluoromount-G (blue). Fluorescence analysis showed the same pattern of infiltration of CD3^+^ and F4/80^+^ predominantly at the tumor front. Dashed line represents tumor–brain intersection. (E–H) Similar immunostaining in the tumor core, showing reduced immunocyte infiltration. (I) Curves representing quantitative image analysis of the immunofluorescence intensity of CD3^+^ and (J) F4/80^+^ cells. The numbers denote mean fluorescence intensity (MFI). Data are representative of three independent experiments (*P* < 0.05). (K) Histogram showing quantification of CD3^+^ cells (mean ± SEM; *n* = 10). (L) Histogram showing quantification of F4/80^+^ cells. All scale bars are 100 μm.

To investigate whether our animal model recapitulates the nature of the human disease, we further investigated the immunocytes infiltration in specimens resected from brains of 10 patients with metastatic melanoma. In agreement with our animal study, analysis of the human specimens revealed that the F4/80-positive cells were more abundant at the tumor front than in its core (*P* = 0.001, Fig. 3L). Similarly, analysis of the T-cell component revealed that the prominent CD3-positive cell infiltrates were at the tumor front compared with the tumor core (*P* = 0.003, Fig. 3K). Validation testing with fluorescent intensity analysis was consistent with cell count, and it revealed stronger staining with anti-CD3 and anti-F4/80 Abs at the tumor front compared with the tumor core (*P* < 0.05, Fig. 3I and J).

### Spatial architecture of brain melanoma metastasis vasculature

**Figure 4:**
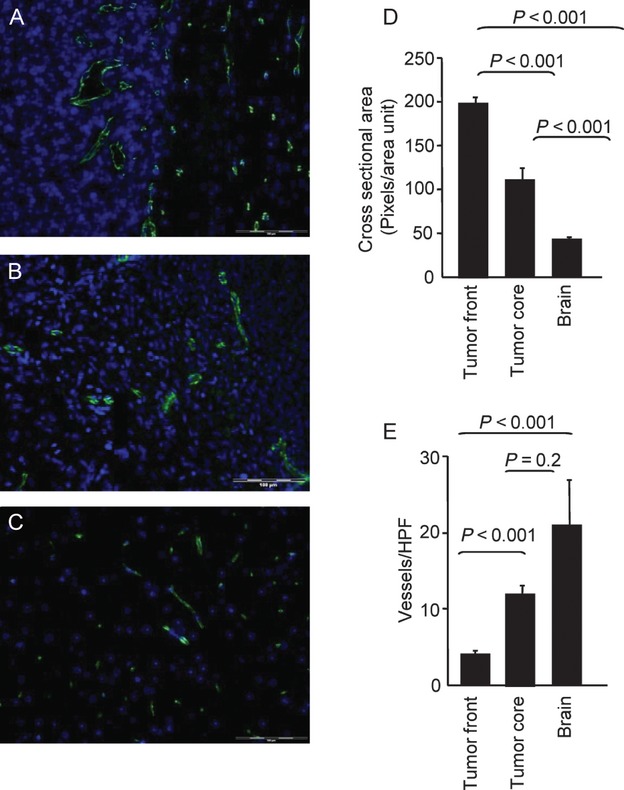
Immunohistochemical analysis of angiogenesis in mice. (A) Immunofluorescence staining with anti-CD31 (green) Ab of tumor front, (B) tumor core, and (C) normal brain. Nuclei were stained with Dapi-Fluoromount-G (blue). The tumor front contained 4 ± 0.6 vessels per high-power field (HPF) compared with 12 ± 1 vessels per HPF within the tumor core (mean ± SEM, *n* = 8; *P* < 0.01). (D) Histograms showing vessel area. (E) Histograms showing vessel number. The microvessel density (MVD) was calculated in each region. The MVD was three times higher within the tumor core compared with the tumor front (*n* = 8; *P* < 0.01). Dashed line represents tumor–brain intersection. All scale bars are 100 μm.

**Figure 5:**
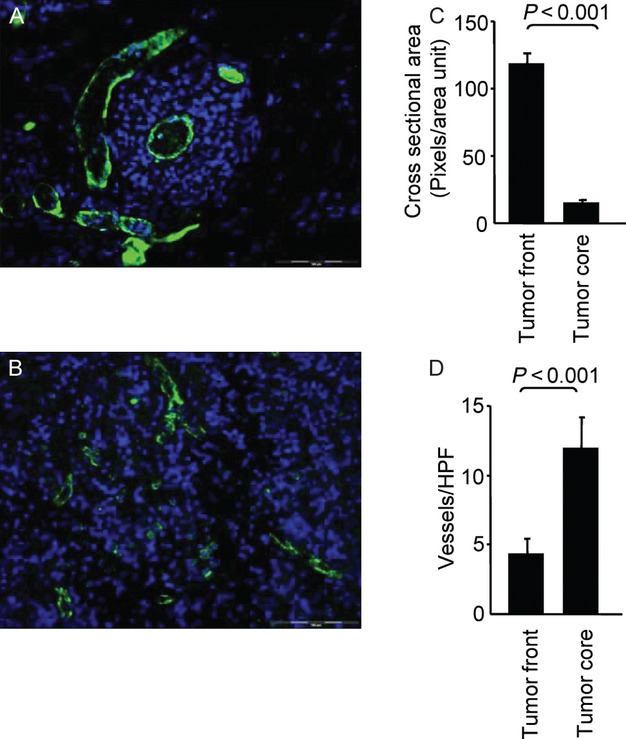
Immunohistochemical analysis of angiogenesis in human. (A) Immunofluorescence staining with anti-CD31 (green) Ab of the tumor front and (B) the tumor core. Nuclei were stained with Dapi-Fluoromount-G (blue). The tumor front contained 4.3 ± 1.1 vessels per high-power field (HPF) compared with 11.9 ± 2.3 vessels per HPF within the tumor core (mean ± SEM, *n* = 10; *P* < 0.001). (C) Histograms showing vessel area. (D) Histograms showing vessel number. The microvessel density (MVD) was calculated in each region. The MVD was 12 times higher within the tumor core compared with the tumor front (*n* = 10; *P* < 0.001). Dashed line represents tumor–brain intersection. All scale bars are 100 μm.

A hallmark of metastatic growth is the formation of new vessels, a process which is influenced by tumor factors as well as by host factors 14. We performed immunohistochemical analysis of the mouse model using anti-CD31 antibody in order to evaluate the spatial architecture of the angiogenesis pattern in our model (Fig. 4). This analysis revealed multiple small vessels within the tumor core (12 ± 1 vessels per HPF, average cross-sectional area 4617 μm^2^, *n* = 8). In contrast, there were few vessels at the tumor front (4 ± 0.6 vessels per HPF, *P* < 0.001, average cross-sectional area 8236 μm^2^), and they were significantly larger than those in the tumor core (*P* < 0.005). The same angiogenesis pattern was found in the specimens of human metastases: it showed fewer vessels at the tumor front than within its core (4.3 ± 1.1 and 11.9 ± 2.3 vessels per HPF, respectively, *P* < 0.001). The mean cross-sectional area for vessels within the tumor core was significantly lower than at the tumor front (624 ± 82 and 4908 ± 328 μm^2^, respectively, *P* < 0.001). Figure 5 shows the analysis of the vascular architecture found in and around the human brain melanoma metastases.

## Discussion

This study describes a GFP-expressing animal model of brain melanoma metastasis and compares its stromal reaction to the human disease. The parameters that we evaluated in this model included the spatial architecture of immunocyte infiltration and the angiogenesis pattern. Both the mouse model and the human tumor share the following features: (1) macrophages/microglia are more abundant at the tumor front than in its core, (2) T-cell infiltration is more prominent at the tumor front, (3) the majority of these lymphocytic cells are CD4 positive, (4) there are fewer vessels at the tumor front than in its core, and (5) the number of vessels is inversely correlated with their diameters.

In experimental mouse models, brain metastases display a 50% higher blood vessel density compared to the primary tumor 15,16. Hyperdilated and proliferating blood vessels are characteristic of high-grade brain tumors and brain metastases in patients as well as in experimental models 17,18. Different mechanisms of neovascularization have been described for brain tumors. These include growth of cancer cells around the preexisting blood vessels (vessel cooption), sprouting of vessels (angiogenesis), and vasculogenesis, which is a recruitment of endothelial progenitor cells that have been proposed to originate from different sources, including the bone marrow or the existing vasculature 19,20. Angiogenesis can be induced by microglia/macrophages that secrete multiple cytokines, growth factors (e.g., vascular endothelial growth factor), enzymes, and reactive oxygen species 6,21–23. Activated F4/80-expressing microglia/macrophages are frequently found to infiltrate primary and metastatic brain tumors in human patients and in animal models of glioma and breast and lung cancer metastasis 6,22–25. In animal models that harbor GFP-labeled bone marrow-derived cells, a significant proportion of the glioma-associated F4/80+ microglia/macrophages has been shown to originate from the newly infiltrating bone marrow-derived monocytes 26. The brain tumor-associated microglia/macrophages seem to be a mixed population derived from the brain-resident microglia and from the newly infiltrating monocytes 27. The correlation between local macrophage density and areas of intense angiogenesis suggests a role for macrophages in this process 28.

Our data on T-cells infiltrates revealed a high CD4:CD8 ratio. A similar pattern was shown to be indicative of poor prognosis in other malignancies, including breast and colorectal carcinoma 29,30–32. For example, Naito et al. demonstrated that the accumulation of CD8+ T cells within the tumor is associated with good outcome, whereas accumulation of the same cells at the tumor front has no effect on survival 33. Others have shown that the ratio and distribution patterns of intratumoral CD8+ T cells and CD4+ T cells were critical determinants of disease progression 30,34–36.

An abundant number of tumor-associated macrophages has also been associated with the dismal clinical course of metastatic melanoma 37. It is also consistent with the clinical data in other carcinomas in showing that macrophage infiltration is a predictor of poor prognosis 38,39.

Our finding of numerous immune cells in the periphery of brain metastases, and not in the tumor core (Figs. 2 and 3), is in agreement with previous findings showing that angiogenesis emerges from vascular remodeling of large vessels by a process called nonsprouting angiogenesis 40,41–43. In this process, new blood vessels are formed by the “splitting” of preexisting dilated blood vessels. This process, which reflects an increased mean vascular density at the periphery of a tumor, is correlated with poor prognosis 44.

We are aware that GFP per se may contribute to the immunogenic reaction against the tumor in our syngeneic melanoma model 45. Yet, the similarity of human immune response in the absence of GFP to the response in its presence in mice makes it least likely. We are aware that an intracardiac injection of cells bypasses the initial steps of metastasis (i.e., separation from the primary neoplasm, invasion, and release into blood vessels). Nevertheless, our findings clearly show that our animal model recapitulates the angiogenesis pattern and immune reaction at the metastatic niche 46. The identification of an in vivo model capable of recapitulating the human form of the disease, as revealed by comparative analysis of murine and human brain specimens, is the first step in the testing of new treatment modalities against melanoma. Our model also enables a more in-depth study of the immune cell function and the unique pattern of tumor angiogenesis in a syngeneic model of melanoma brain metastases.

## Supporting Information

Figure S1 Tumorigenicity profile of a *B16-GFP* melanoma in C57BL/6 mice. (A) A survival curve of tumor-bearing mice. The median survival time after the intracardiac injection was 26 days for 2 × 10^4^ cells injected, and 29 days for 10^5^ cells injected (*P* = 0.8). Melanoma cells (2 × 10^4^ or 10^5^) were injected under ultrasound guidance into the left ventricle of C57BL/6 mice (*n* = 20). Mice were monitored twice weekly after the inoculation, euthanized when moribund, and necropsied. (B) Organs with metastatic spread. The presence of metastases in anticipated organs was recorded during necropsy.
