# Supplementary material for: Characterization of the melanoma brain metastatic niche in mice and humans
Source: Cancer Med. 2013 Mar 11;2(2):155–63. doi: 10.1002/cam4.45 (PMC3639654; doi:10.1002/cam4.45)
Supplement: Supplementary file 1 [file cam40002-0155-sd1.docx]

A

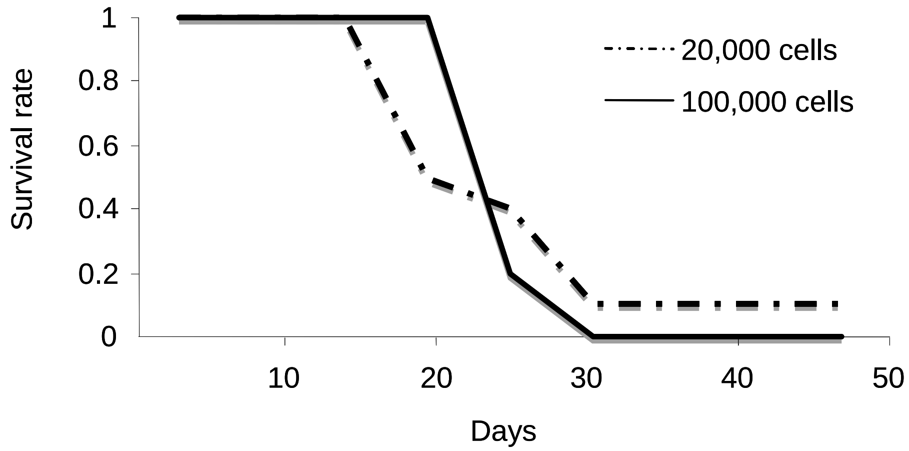

1
0.8
0.6
0.4
0.2
0
Survival rate
20,000 cells
100,000 cells
10
20
30
40
50
Days

B

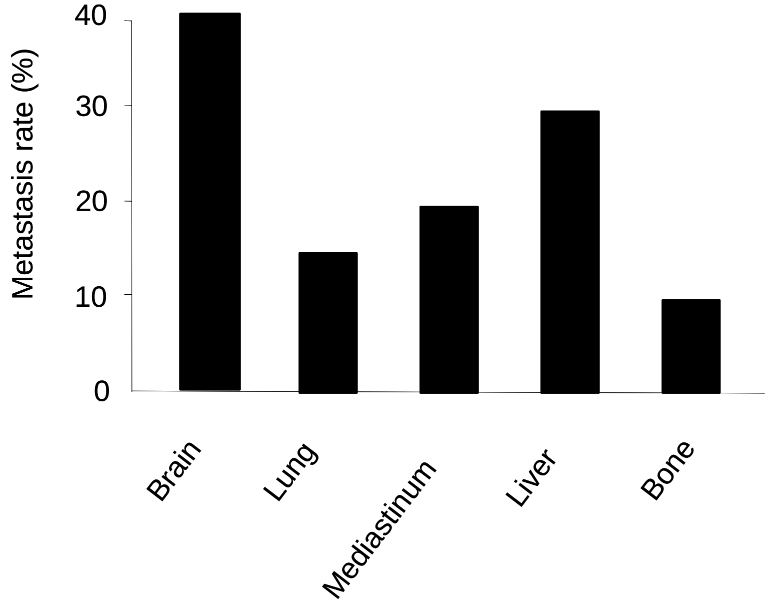

40
30
20
10
0
Metastasis rate (%)
Brain
Lung
Mediastinum
Liver
Bone
